# Artificial intelligence in osteoporosis screening and fracture risk prediction among aging populations: a bibliometric evidence map

**DOI:** 10.3389/fendo.2026.1899276

**Published:** 2026-08-04

**Authors:** Bo Wang, Sijin Tang, Pingjuan Tan, Baohong Mi, Weiheng Chen, Chunzhu Gong, Renxuan Yang, Qiushi Wei

**Affiliations:** 1 Shenzhen PingLe Orthopedic Hospital (Shenzhen Pingshan Traditional Chinese Medicine Hospital), Shenzhen, China; 2 Shenzhen PingLe Orthopedic Hospital Affiliated to Guangzhou University of Chinese Medicine, Shenzhen, China; 3 The Seventh Affiliated Hospital, Sun Yat-sen University, Shenzhen, China; 4 Institute of Orthopedics, Beijing University of Chinese Medicine, Beijing, China; 5 Engineering Research Center of the Ministry of Education for Intelligent Traditional Chinese Medicine Orthopedic Treatment and Sports Rehabilitation, Beijing, China; 6 The Third Affiliated Hospital of Beijing University of Chinese Medicine, Beijing, China

**Keywords:** artificial intelligence, osteoporosis screening, fracture risk prediction, aging populations, bone health

## Abstract

**Objective:**

To map the development, knowledge structure, and clinical translation of artificial intelligence applications in osteoporosis screening and fracture risk prediction among aging populations, with a focus on risk stratification, prediction-oriented modeling, and endocrine bone health management.

**Methods:**

A bibliometric evidence-mapping study was conducted using publications retrieved from the Web of Science Core Collection and PubMed. VOSviewer and R/bibliometrix were used to analyze publication and citation trends, collaboration networks, reference co-citation knowledge bases, author keyword structures and bursts, keyword–knowledge-base consistency, and interdisciplinary knowledge-flow patterns at the discipline and journal levels.

**Results:**

A total of 371 publications were included. Among 10,934 cited references, 74 met the co-citation threshold and formed four major knowledge-base clusters related to early fracture risk assessment and epidemiological evidence, fracture risk model construction, guideline-based osteoporosis management, and machine-learning expansion. Author keyword analysis included 70 keywords and identified four application-oriented themes: aging-related osteoporosis and fracture-risk screening frameworks, machine-learning risk stratification in older fracture-related populations, imaging and radiomics markers of structural fragility, and deep-learning applications for prediction-oriented detection and triage. Cross-mapping between keyword themes and co-citation clusters showed that most current hotspots were supported by established fracture-risk, BMD, FRAX, guideline, or machine-learning knowledge bases, whereas deep-learning-related themes remained more distributed across clinical and methodological clusters. Annual publications increased from 15 in 2019 to 86 in 2025. China and the United States were the leading contributors, with 126 and 90 publications, respectively. Knowledge-flow analyses showed increasing input from Radiology & Imaging, Computer & AI, and Engineering, with strong convergence toward Clinical Medicine and continued aggregation in core osteoporosis and bone metabolism journals.

**Conclusion:**

This study provides a population-specific evidence map of AI applications in osteoporosis screening and fracture risk prediction among aging populations. The findings suggest that this field is moving from conventional risk assessment toward AI-assisted risk stratification, imaging-based screening, and clinically oriented decision support. Future research should prioritize aging-specific validation, multimodal integration, interpretability, fairness, external validation, and real-world evaluation before AI-based fracture risk tools can be routinely incorporated into endocrine and bone health practice.

## 1 Introduction

Population aging has made bone health management an increasingly important public health priority. In middle-aged and older adults, osteoporosis, fragility fractures, hip fractures, falls, sarcopenia, and multimorbidity often interact with each other, creating a long-term risk continuum from bone loss and structural fragility to fracture occurrence, postoperative complications, disability, and mortality. Osteoporotic fractures, especially hip and vertebral fractures, can substantially impair quality of life and impose a sustained burden on health systems, families, and long-term care services (1). Therefore, earlier identification of individuals at increased fracture risk, timely osteoporosis or fracture-risk screening, and prediction-oriented management have become central issues in aging-related bone health and public health research (2).

Fracture risk assessment is an essential component of aging-related bone health management. Traditional approaches, including bone mineral density measurement and FRAX-based tools, have played important roles in osteoporosis screening, fracture-risk stratification, and clinical decision-making (3, 4). However, as the field has advanced, limitations have become apparent in the integration of multiple risk factors, individualized prediction, and the modeling of complex nonlinear relationships. Fracture risk in the elderly is influenced by various factors, including age, sex, bone mineral density, previous fractures, falls, comorbidities, medication exposure, lifestyle factors, and imaging-derived structural features, making it difficult for single-factor or strictly linear models to capture individual risk profiles accurately (5). In recent years, artificial intelligence (AI) technologies, especially machine learning and deep learning methods, have been widely applied in medical risk prediction and clinical decision support (6). Previous studies suggest that AI methods may be well suited to high-dimensional, multivariate, and nonlinear data, offering new opportunities to improve fracture risk prediction and support personalized assessment (7, 8). Research on AI-based fracture risk prediction is increasing, covering model construction, feature selection, and multi-source data integration, showing rapid development (9). Recent reviews have further summarized the expanding role of AI in osteoporosis diagnosis and management. These studies indicate that AI and machine-learning approaches are increasingly being applied to osteoporosis diagnosis, bone mineral density assessment, imaging-based screening, fracture risk prediction, and clinical decision support, and they also highlight the growing use of X-ray, DXA, CT, MRI, radiomics, deep learning, and multimodal data integration in osteoporosis-related applications (10–12).

However, despite these recent application-oriented reviews, the development of AI in osteoporosis screening and fracture risk prediction among aging populations remains insufficiently characterized from a knowledge-structure perspective. In particular, it remains unclear how current AI applications are supported by established knowledge bases in fracture risk assessment, BMD, FRAX, clinical guidelines, imaging-based assessment, and machine-learning methods, and how knowledge circulates across clinical medicine, radiology and imaging, engineering, and computer science. To address these gaps, the present study provides a population-specific and application-oriented bibliometric evidence map of AI applications in osteoporosis screening and fracture risk prediction among middle-aged and older adults. We incorporated both the Web of Science Core Collection and PubMed to strengthen bibliometric and biomedical coverage, and further extended the analysis beyond publication trends and keyword hotspots to reference co-citation knowledge bases, keyword–knowledge-base consistency, theme-to-discipline diffusion, discipline-to-discipline flows, discipline-to-journal flows, and disciplinary diversity. By integrating these analytical layers, this study aims to clarify the knowledge foundations, emerging application pathways, and translational challenges of AI-assisted fracture risk prediction in endocrine bone health and aging-related clinical practice.

## 2 Materials and methods

### 2.1 Data sources and search strategy

The bibliographic data were retrieved from the Web of Science Core Collection (WoSCC) and PubMed databases. WoSCC was selected as the primary bibliometric source because it provides standardized citation metadata, cited-reference information, author affiliations, journal categories, and institutional information required for co-citation, collaboration, and knowledge-flow analyses. PubMed was incorporated to strengthen biomedical and clinical coverage because it has strong coverage of clinically relevant biomedical literature and controlled biomedical indexing. This database combination was chosen to support both bibliometric network construction and biomedical relevance. The literature search and export were conducted on January 14, 2026, with the database time limit set to records published up to December 31, 2025.

In WoSCC, an advanced search was performed using the following strategy: TS=((osteoporosis OR “bone fracture” OR “fragility fracture” OR “hip fracture*”) AND (“fracture risk” OR prediction OR prognos* OR “risk model*” OR “risk assessment”) AND (“artificial intelligence” OR “machine learning” OR “deep learning” OR algorithm* OR “neural network*” OR “random forest” OR “support vector machine”) AND (aging OR elderly OR “older adult*” OR geriatric* OR age*)), with the restrictions Document Types = Article or Review and Language = English.

In PubMed, the search strategy was as follows: (“osteoporosis” OR “bone fracture”[Title/Abstract] OR “fragility fracture”[Title/Abstract] OR “hip fracture”[Title/Abstract]) AND (“fracture risk”[Title/Abstract] OR prediction[Title/Abstract] OR prognos*[Title/Abstract] OR “risk model”[Title/Abstract] OR “risk assessment”[Title/Abstract]) AND (“artificial intelligence”[MeSH Terms] OR “machine learning”[MeSH Terms] OR “deep learning”[Title/Abstract] OR “algorithm*”[Title/Abstract] OR “neural network*”[Title/Abstract] OR “random forest”[Title/Abstract] OR “support vector machine”[Title/Abstract]) AND (“aging”[MeSH Terms] OR “elderly”[MeSH Terms] OR “older adult*”[Title/Abstract] OR “geriatric*”[MeSH Terms] OR “age*”[MeSH Terms]). MeSH terms were used where appropriate, while other terms were searched as Title/Abstract terms to capture recent, method-specific, or non-indexed terminology. Therefore, not all search terms were intended to be MeSH terms.

To harmonize the two database searches, the WoSCC and PubMed strategies were organized around the same four conceptual blocks: osteoporosis or fracture-related conditions, fracture risk assessment or prediction, AI-related methods, and aging-related populations. In WoSCC, Topic Search was used because it covers titles, abstracts, author keywords, and Keywords Plus, which are commonly used for bibliometric retrieval. In PubMed, MeSH terms were used for established biomedical concepts when available, and Title/Abstract terms were used for recent, method-specific, or non-indexed expressions, especially AI-related terms. All records retrieved from both databases were subsequently deduplicated and screened using the same predefined eligibility criteria. The conceptual harmonization of the two search strategies is summarized in **Supplementary Table 1**.

### 2.2 Eligibility criteria, screening, and data cleaning

The search yielded 819 records from WoSCC and 63 records from PubMed, giving 882 records before deduplication. The screening process was designed to construct a focused analytical sample relevant to aging-related bone health. The target population was middle-aged and older adults or populations relevant to aging-related bone health. The analytical scope covered AI-related fracture risk assessment, osteoporosis or fracture-risk screening, and future fracture prediction in clinical, imaging, epidemiological, population-level, or health data settings. The inclusion criteria were as follows: (1) studies focusing on fracture risk prediction, risk assessment, or osteoporosis/fracture-risk screening; and (2) studies in which the target population or application scenario mainly involved middle-aged and older adults. The exclusion criteria were: (1) publication types other than articles or reviews, such as meeting abstracts, editorials, letters, corrections, or book reviews; (2) retracted publications or duplicate records; (3) studies without AI-related methods, algorithmic modeling, machine learning, deep learning, or computational prediction; and (4) studies outside the scope of fracture risk assessment, osteoporosis or fracture-risk screening, future fracture prediction, or fracture-related risk assessment in middle-aged and older adults. Studies focusing solely on acute fracture detection without relevance to risk assessment, screening, or prediction-oriented evaluation were also excluded. Conventional risk tools, including FRAX-related studies, were retained only when they provided foundational evidence, were integrated with AI-related approaches, or served as comparators in prediction-oriented studies. Postoperative outcome studies were included only when they involved AI-based risk prediction in older fracture patients.

**Figure 1:**
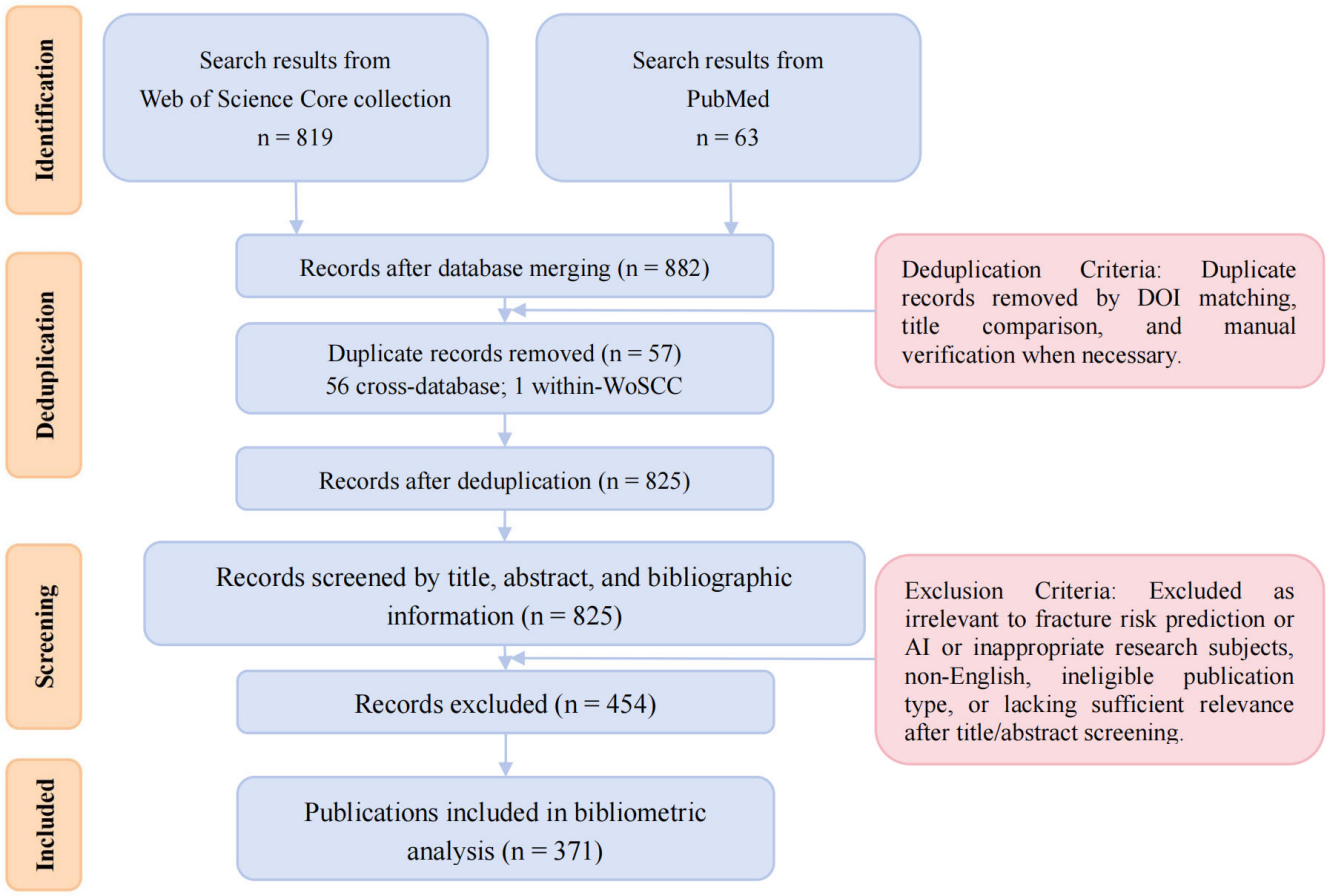
Flowchart of literature search and screening process.

Two trained reviewers independently screened titles, keywords, and abstracts, and full texts or extended bibliographic records were examined when eligibility was uncertain. Disagreements were resolved through discussion, and unresolved cases were adjudicated by a senior author. Records retrieved from WoSCC and PubMed were imported into R and merged into a single screening dataset. Deduplication was performed in a stepwise manner using DOI and PMID when available, followed by manual verification based on normalized title, first author, publication year, and journal name. The initial search retrieved 819 records from WoSCC and 63 records from PubMed, yielding 882 records before deduplication. We identified 56 cross-database duplicates between WoSCC and PubMed and 1 within-WoSCC duplicate record. After removing these 57 duplicate records, 825 unique records remained for eligibility screening. Among the PubMed records, 7 were PubMed-only records after deduplication. These records were excluded during eligibility screening because they did not meet the predefined inclusion criteria for AI-related osteoporosis screening or fracture risk prediction in middle-aged and older adults. Therefore, PubMed served as a complementary biomedical source for retrieval verification, while the final included records were all represented in WoSCC. Ultimately, 371 publications were included in the final bibliometric analysis. The database overlap, deduplication process, and source-specific screening results are summarized in **Figure 1**; **Supplementary Table 2**.

### 2.3 Bibliometric and visualization analysis

The study employed VOSviewer, version 1.6.20 (Centre for Science and Technology Studies, Leiden University, Leiden, the Netherlands), and R, version 4.5.1 (R Foundation for Statistical Computing, Vienna, Austria), with the bibliometrix package, version 4.5.1, for bibliometric and visualization analyses. VOSviewer was used mainly for network construction and visualization, including country, institution, author, reference co-citation, and author keyword co-occurrence networks. R and the bibliometrix package were used for data cleaning, descriptive bibliometric statistics, annual publication and citation trends, burst-related summaries, discipline/journal-level flow matrices, and diversity calculations. The combined use of these tools allowed network visualization and quantitative bibliometric summaries to complement each other. VOSviewer default counting and clustering settings were used unless otherwise specified. Thresholds were selected to balance network interpretability and information retention. The analysis was designed as a bibliometric evidence-mapping workflow rather than a general hotspot-only analysis. The main analyses included:

1. Population-specific research activity and collaboration structure: annual publication and citation trends were calculated, and collaboration networks of countries/regions, institutions, and authors were constructed to characterize the overall development of the field.
2. Co-citation knowledge-base mapping: a reference co-citation network was constructed with a minimum citation threshold of 10. Structural indicators, including total link strength (TLS), number of links, average citation count, and average publication year, were calculated. Cluster analysis and temporal overlay analysis were used to identify core knowledge modules, their structural characteristics, and their temporal evolution. In addition, stage-specific thematic distribution was summarized using keyword dictionary matching based on titles, author keywords, Keywords Plus, and abstracts. For the reference co-citation analysis, a minimum citation threshold of 10 was used in the main analysis. This threshold was selected to balance network interpretability and retention of a sufficiently connected core knowledge base. A lower threshold would retain more cited references but generate a denser network, whereas a higher threshold would focus on a smaller set of highly co-cited references and might omit relevant but less frequently cited studies. To examine whether the main knowledge-base structure was dependent on this threshold, we performed sensitivity analyses using alternative thresholds of 8 and 12 citations for cited references. The stability of the co-citation structure was assessed by comparing the number of retained cited references, links, total link strength, number of clusters, and thematic composition across thresholds. The results are summarized in **Supplementary Table 3**.
3. Hotspot–knowledge-base consistency analysis: an author keyword co-occurrence network was generated using a minimum occurrence threshold of 3, followed by cluster analysis to identify major thematic domains. Cluster labels were assigned through a consensus-based interpretive process. Three authors reviewed the high-frequency terms, representative nodes, and highly co-cited references within each cluster, and determined the final labels based on bibliometric evidence and domain knowledge. Keyword burst analysis was further performed to detect emerging topics and recent shifts in research attention. Keyword clusters were also compared with co-citation clusters to examine whether major hotspots were supported by identifiable knowledge bases. Before author keyword co-occurrence analysis, author keywords were normalized using a predefined thesaurus and manual verification procedure. The normalization process included lowercasing, removal of redundant punctuation and extra spaces, harmonization of hyphenated and non-hyphenated forms, singular/plural unification, abbreviation expansion, and merging of clear synonyms or spelling variants. For example, hyphenated variants such as “machine-learning” were normalized as “machine learning”; “BMD” and “bone mineral density (BMD)” were normalized as “bone mineral density”; “DXA” was normalized as “dual-energy X-ray absorptiometry”; and “prediction models,” “predictive model,” and “predictive models” were normalized as “prediction model”. Non-thematic terms introduced during export or parsing were removed. Terms were merged only when they referred to the same concept or to a clearly equivalent variant of the same concept. The keyword normalization dictionary is provided in **Supplementary Table 4**.
4. Interdisciplinary and journal-level diffusion mapping: based on citing literature, the temporal distribution of citing disciplines was analyzed, and flow structures were constructed at the theme-to-discipline, discipline-to-discipline, and discipline-to-journal levels. Core flow paths and the Shannon diversity index were used to quantify interdisciplinary knowledge diffusion and integration.

## 3 Results

### 3.1 Population-specific research activity and collaboration structure

#### 3.1.1 Publication growth trends and stage-based development

**Figure 2:**
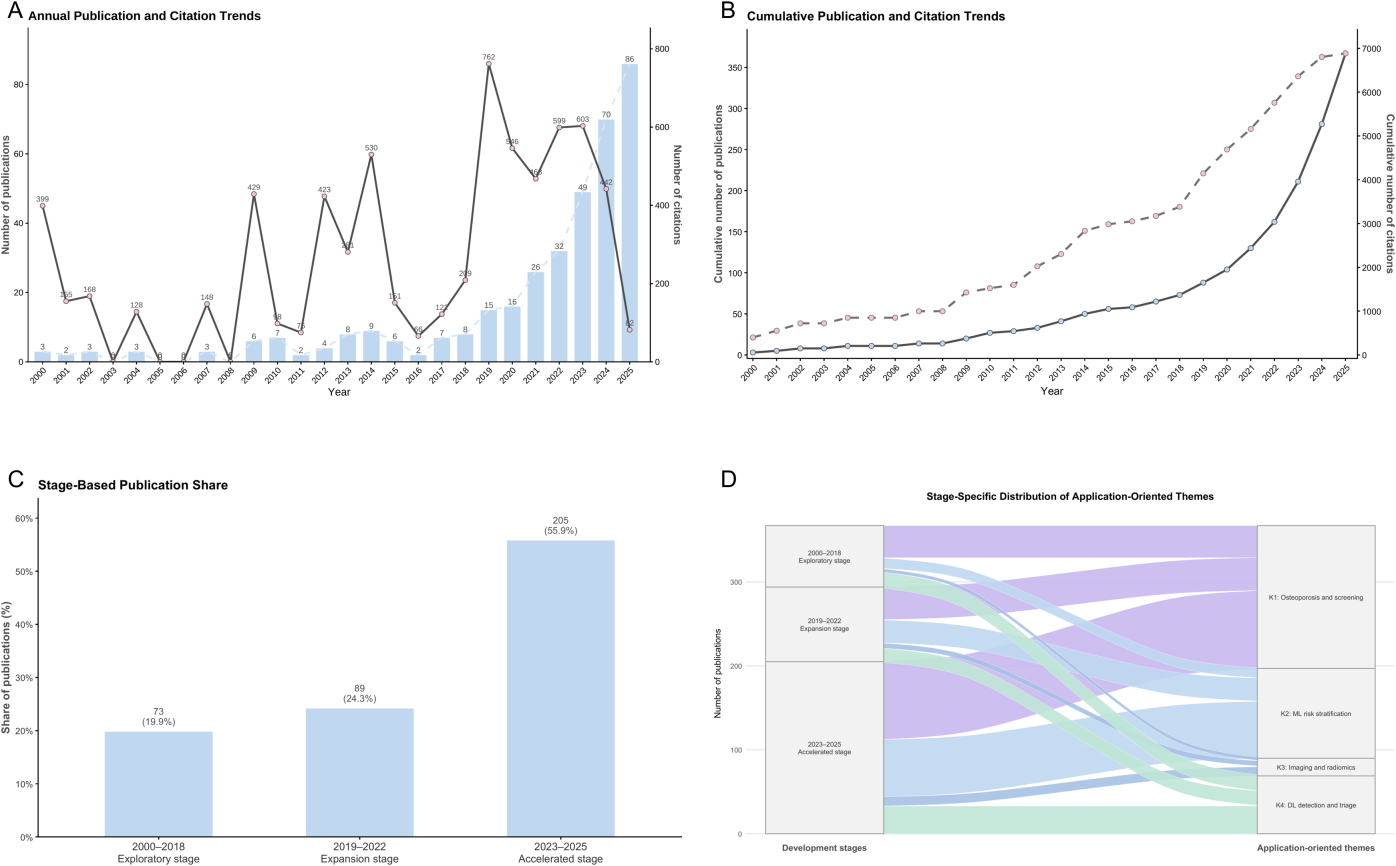
Temporal growth and stage-specific thematic distribution of the field. **(A)** Annual publication and citation trends, showing yearly research output and citation activity. **(B)** Cumulative publication and citation trends, illustrating the overall expansion of the field over time. **(C)** Stage-based publication share across the exploratory, expansion, and accelerated stages, showing the relative contribution of each developmental period. **(D)** Stage-specific distribution of application-oriented themes. **(D)** was generated using keyword dictionary matching based on titles, author keywords, Keywords Plus, and abstracts, and was used to describe how major application themes were distributed across different developmental stages.

Annual publication and citation trends showed an overall increase in AI-related fracture risk assessment, screening, and prediction among middle-aged and older adults (**Figure 2A**). From 2000 to 2018, annual output generally remained below 10 publications, indicating an exploratory phase. Since 2019, publication output increased from 15 papers to 32 in 2022, 49 in 2023, 70 in 2024, and 86 in 2025, suggesting a phase of accelerated expansion. Annual citation counts fluctuated but remained relatively high after 2019, with the highest value observed in 2019 (762 citations).

The cumulative curves further showed a clear acceleration of both publication output and citation accumulation after 2019 (**Figure 2B**). Stage-based analysis showed that recent years contributed the largest share of publications, with the 2023–2025 accelerated stage accounting for the largest proportion of total output (**Figure 2C**). The stage-specific thematic distribution further suggested that application-oriented themes became more visible and diversified during the later stages, especially themes related to machine-learning risk stratification, imaging and radiomics markers, and deep-learning-assisted prediction-oriented detection or triage (**Figure 2D**). Overall, **Figure 2** indicates that this population-specific field has moved from a low-output exploratory stage toward a more active and thematically diversified phase.

#### 3.1.2 Major country/region contributions and collaboration patterns

**Figure 3:**
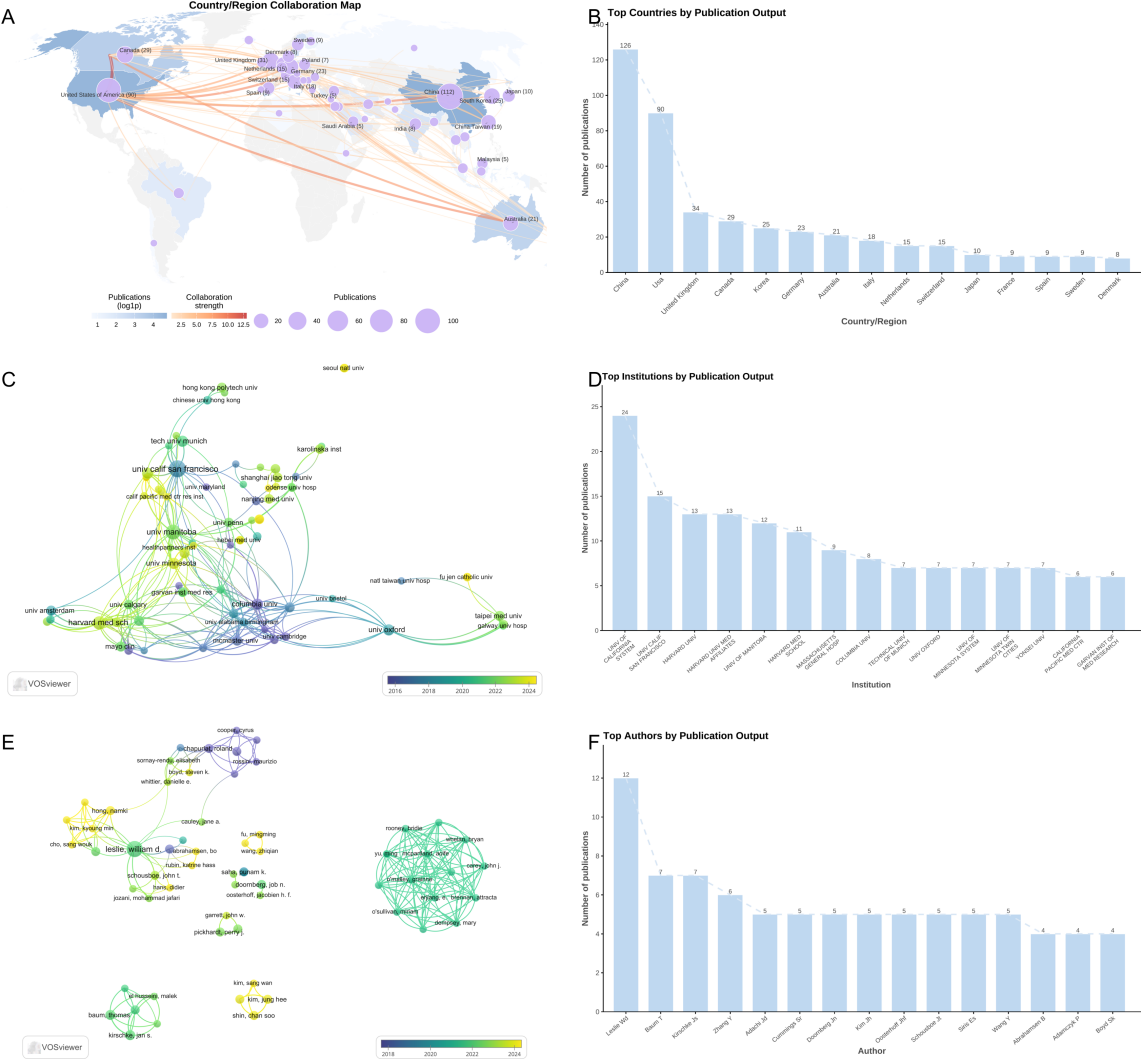
Research output and collaboration patterns at the country/region, institutional, and author levels. **(A)** Global country/region collaboration map. **(B)** Top countries/regions by publication output. **(C)** Institutional collaboration network visualized by VOSviewer. **(D)** Top institutions by publication output. **(E)** Author collaboration network visualized by VOSviewer. **(F)** Top authors by publication output. Together, these panels illustrate the geographic distribution of research productivity and the collaborative structure of the field across multiple levels.

Country/region-level analysis showed a concentrated but internationally connected research landscape (**Figures 3A, B**). China ranked first with 126 publications, followed by the United States with 90. The United Kingdom, Canada, South Korea, Germany, Australia, Italy, the Netherlands, and Switzerland also contributed visible output. The collaboration map showed that China and the United States occupied central positions and were connected with multiple countries across Asia, Europe, and Oceania. European countries displayed relatively dense intra-regional linkages. Overall, research output was concentrated in a limited number of productive countries/regions, while collaboration was organized around several major international hubs.

#### 3.1.3 Institutional and author collaboration structures

At the institutional level, publication output was also concentrated in a limited number of organizations (**Figures 3C, D**). The University of California System ranked first with 24 publications, followed by the University of California, San Francisco (15), Harvard University (13), Harvard University Medical Affiliates (13), the University of Manitoba (12), and Harvard Medical School (11). The institutional collaboration network showed multiple interconnected subgroups rather than a single dominant center, indicating that collaboration was organized through several active institutional clusters.

At the author level, a similar concentration pattern was observed (**Figures 3E, F**). Leslie WD was the most productive author with 12 publications, followed by Baum T and Kirschke JS with 7 publications each, and Zhang Y with 6 publications. The author collaboration network revealed multiple relatively independent research groups, with dense internal collaboration within clusters but comparatively limited links between clusters. Overall, institutional and author collaborations were organized through several semi-connected communities, indicating that research capacity in this focused area remains distributed across multiple active groups rather than a single dominant center.

### 3.2 Co-citation knowledge bases and hotspot–knowledge-base consistency

#### 3.2.1 Reference co-citation structure and core knowledge modules

**Figure 4:**
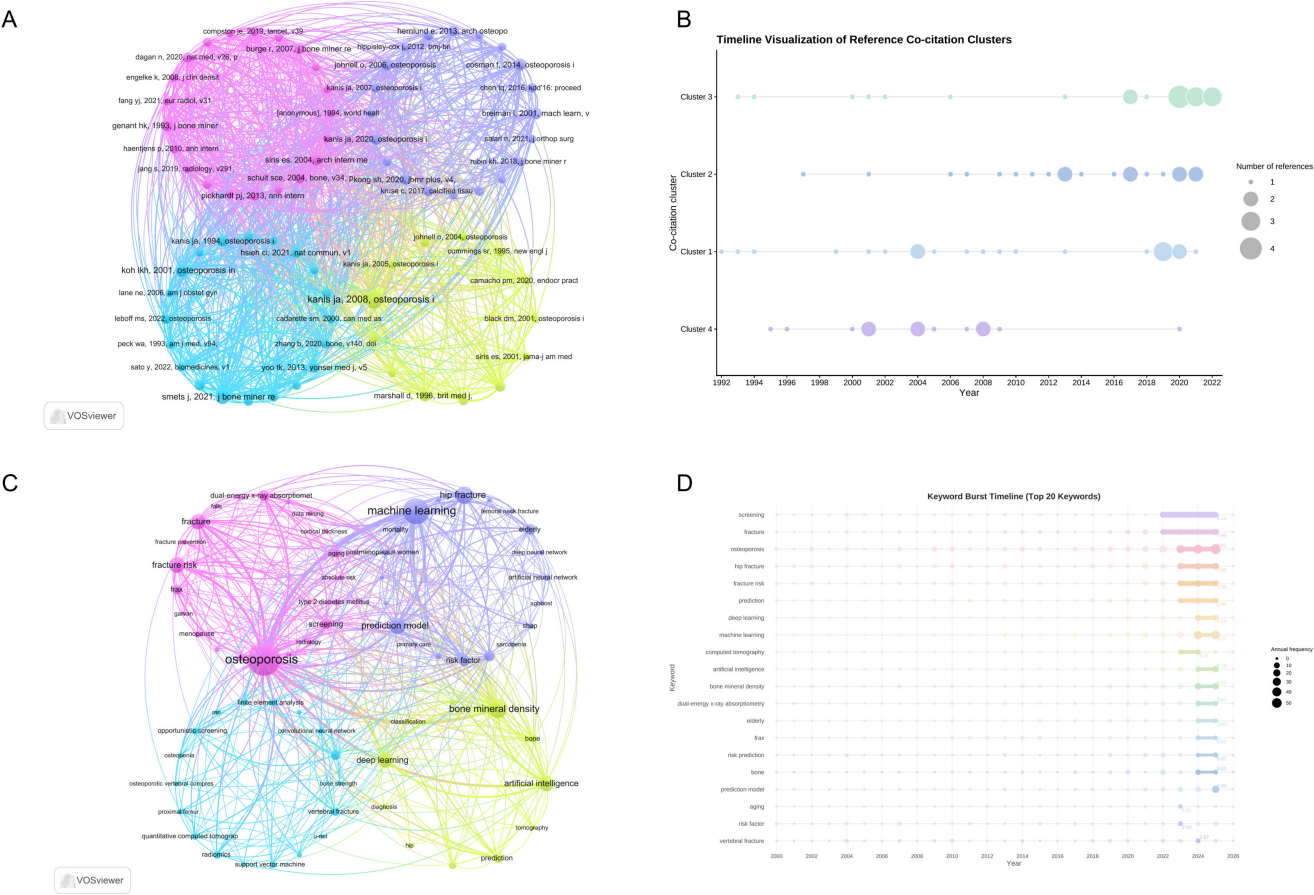
Knowledge base structure, thematic hotspots, and temporal evolution. **(A)** Reference co-citation network showing the core knowledge base and four major co-citation clusters. **(B)** Temporal overlay of the reference co-citation network, illustrating the chronological distribution and evolution of major knowledge modules. **(C)** Author keyword co-occurrence network showing four major thematic clusters and their semantic relationships. **(D)** Keyword burst analysis highlighting emerging topics and shifting research attention over time.

To move beyond descriptive hotspot mapping, we first examined the co-citation structure of cited references to identify the knowledge bases supporting AI-related fracture risk assessment, screening, and prediction in middle-aged and older adults. Among 10,934 cited references, 74 met the threshold of at least 10 citations, accounting for 0.68% of the total and forming the core reference co-citation network (**Figure 4A**, **Table 1**). The network contained 74 nodes and 1,386 links, with a total link strength (TLS) of 2,478, indicating a compact and highly interconnected knowledge base. To assess the robustness of this threshold-dependent network structure, we performed sensitivity analyses using alternative citation thresholds of 8 and 12 for cited references. When the threshold was relaxed to 8, 110 cited references were retained, forming 2,349 links with a total link strength of 3,852 and four clusters. When the threshold was tightened to 12, 49 cited references were retained, forming 703 links with a total link strength of 1,381 and four clusters. Across these thresholds, the number of clusters remained unchanged, and the main knowledge-base modules were broadly retained, including fracture-risk assessment and FRAX-related evidence, BMD and structural fragility evidence, guideline-based osteoporosis management, and machine-learning-oriented methodological expansion. These results suggest that the four-module interpretation of the main co-citation network was not solely driven by the threshold of 10 citations.

**Table 1:** Structural characteristics of reference co-citation clusters.

Cluster	Docs (n)	Share (%)	Mean Cit.	TLS	Links	Mean year	Representative reference
C1	21	28.38	13.38	1289	764	2008.4	Siris ES, 2004 (13)
C2	20	27.03	14.6	1197	720	2013.1	Cosman F, 2014 (14)
C3	20	27.03	15.4	1544	802	2013.5	Smets J, 2021 (15)
C4	13	17.57	16.31	926	486	2004.5	Kanis JA, 2008 (16)

Based on the reference co-citation network generated by VOSviewer. Docs, number of documents; Share, proportion in the total network; Mean Cit., average citations per reference; TLS, total link strength; Links, total number of links; Mean Year, average publication year. C1 represents an early risk assessment and epidemiological knowledge base; C4 is the earliest foundational cluster; C2 and C3 indicate more recent development toward guideline integration and machine learning; C3 has the highest TLS, suggesting a relatively central position in the knowledge structure.

Cluster analysis identified four major co-citation modules: C1 (21 references, 28.38%), C2 (20 references, 27.03%), C3 (20 references, 27.03%), and C4 (13 references, 17.57%) (**Table 1**). C3 had the highest TLS (1,544) and the largest number of links (802), indicating the strongest structural centrality in the network. C4 had the highest mean citation count (16.31) and the earliest mean publication year (2004.5), suggesting that it represented an earlier and highly influential knowledge component despite its smaller size. By contrast, C2 and C3 had more recent mean publication years (2013.1 and 2013.5, respectively), indicating their later consolidation within the field.

The four clusters showed distinct thematic characteristics. C4 mainly represented the early foundation of fracture risk assessment and epidemiological evidence, including studies on osteoporosis burden and early risk stratification. C1 centered on fracture probability estimation and risk model construction, especially the FRAX-related knowledge base. C2 focused on guideline-based management and standardized clinical frameworks, reflecting the integration of risk assessment evidence into clinical practice. C3 represented the machine learning and AI-oriented module, including methodological foundations and their application in osteoporosis and fracture prediction. Overall, the co-citation structure indicates that current AI-related research in this population is embedded in a layered knowledge base, extending from epidemiological and fracture-risk assessment foundations to guideline-based management and, more recently, machine-learning-oriented methodological expansion.

#### 3.2.2 Core highly co-cited references and knowledge foundations

The top 10 highly co-cited references represented the main intellectual anchors of the field (**Table 2**). These references were concentrated in four functional areas: fracture risk assessment frameworks and FRAX development, BMD and structural indicators for fracture prediction, epidemiological burden and population-level risk evidence, and methodological foundations for machine learning applications. Together, they link conventional fracture-risk assessment, osteoporosis screening, clinical management, and emerging AI methods within a shared knowledge base.

**Table 2:** Top 10 co-cited references and thematic classification.

No.	Author	Year	Journal	Type	Contribution	Theme
1	Kanis JA	1994	Osteoporos Int	WHO Report/Risk Assessment Framework	Proposed a fracture risk assessment and screening framework, laying the theoretical foundation for FRAX.	Foundation of Fracture Risk Assessment Model
2	Marshall D	1996	BMJ	Meta-analysis	Systematic review of BMD’s predictive ability for fractures.	BMD Predictive Effectiveness Evidence
3	Koh LK	2001	Osteoporos Int	Risk Screening Tool	Proposed the OSTA tool for osteoporosis risk screening in Asian women.	Simplified Screening Tool
4	Breiman L	2001	Mach Learn	Methodological study	Introduced the Random Forest algorithm, a foundational machine-learning method widely used in predictive modeling.	Methodological foundation of machine learning
5	Johnell O	2006	Osteoporos Int	Epidemiology	Estimated the global prevalence and burden of osteoporotic fractures.	Epidemiological Burden
6	Kanis JA	2008	Osteoporos Int	Risk Prediction Model	Proposed the FRAX model for fracture probability assessment in both men and women.	FRAX Model Development
7	Schuit SC	2004	Bone	Cohort Study	Assessed the correlation between BMD and fracture occurrence in the Rotterdam Study.	Cohort Validation Study
8	Cosman F	2014	Osteoporos Int	Clinical Guidelines	NOF clinical prevention and treatment guidelines.	Clinical Management Guidelines
9	Kanis JA	2019	Osteoporos Int	European Guidelines	Updated European guidelines for postmenopausal osteoporosis management.	International Clinical Guidelines Update
10	Smets J	2021	J Bone Miner Res	Review	Review of machine learning applications in osteoporosis.	AI Integration in Osteoporosis

This table presents the top 10 co-cited references identified in this study. Type = study type; Contribution = main content or contribution; Theme = thematic classification. The most highly co-cited references mainly focus on fracture risk assessment model development (e.g., FRAX), validation of bone mineral density (BMD) for fracture prediction, assessment of the epidemiological burden of osteoporosis, and development of clinical guidelines, while also covering the methodological basis of machine learning and its application in osteoporosis research. The thematic classifications of all top co-cited references were rechecked in the revised manuscript. Breiman (2001) (17) was classified as a methodological foundation of machine learning because it introduced the Random Forest algorithm.

#### 3.2.3 Keyword co-occurrence structure and thematic distribution

Using a minimum keyword occurrence threshold of 3 and a minimum cluster size of 5, 70 author keywords were included in the co-occurrence analysis, yielding 466 links and a total link strength of 1,247 (**Figure 4C**, **Table 3**). The keyword network formed four major thematic clusters, denoted K1 to K4.

**Table 3:** Author keyword co-occurrence clusters.

Cluster	Theme	Keywords
K1	Aging-related osteoporosis and fracture-risk screening frameworks	absolute risk, aging, cortical thickness, data mining, diabetes, dual-energy x-ray absorptiometry, epidemiology, falls, fracture, fracture prevention, fracture risk, frax, garvan, menopause, nhanes, nomogram, osteoporosis, prevention, radiology, screening, type 2 diabetes mellitus
K2	Machine-learning risk stratification in older fracture-related populations	artificial neural network, deep neural network, elderly, femoral neck fracture, fragility fracture, hip fracture, logistic regression, machine learning, mortality, postmenopausal women, postoperative delirium, postoperative pneumonia, prediction model, primary care, random forest, risk factor, risk prediction, sarcopenia, shap, xgboost
K3	Imaging and radiomics markers of structural fragility	bone strength, computed tomography, convolutional neural network, cortical bone, finite element analysis, fracture prediction, mri, opportunistic screening, osteopenia, osteoporotic vertebral compression fracture, proximal femur, quantitative computed tomography, radiomics, spine, support vector machine, texture analysis, u-net, vertebral fracture
K4	Deep-learning applications for prediction-oriented detection and triage	artificial intelligence, bone, bone mineral density, chest x-ray, classification, deep learning, diagnosis, hip, osteoporotic fractures, prediction, tomography

This table summarizes the results of the author keyword co-occurrence analysis. A minimum keyword occurrence threshold of 3 and a minimum cluster size of 5 were applied, and the network was partitioned using the default clustering algorithm in VOSviewer, yielding four major thematic clusters (K1–K4). Clus., cluster. Theme labels were derived from the core high-frequency keywords within each cluster, and the listed keywords represent representative high-frequency or structurally central terms. The four clusters mainly correspond to aging-related osteoporosis and fracture-risk screening frameworks, machine-learning risk stratification in older fracture-related populations, imaging and radiomics markers of structural fragility, and deep-learning applications for prediction-oriented detection and triage. Keywords were analyzed after normalization using the dictionary provided in **Supplementary Table 4**.

K1 represented aging-related osteoporosis and fracture-risk screening frameworks, characterized by terms related to osteoporosis, fracture risk, FRAX, dual-energy X-ray absorptiometry, screening, menopause, falls, epidemiology, and other clinical variables. K2 reflected machine-learning risk stratification in older fracture-related populations, with keywords such as machine learning, prediction model, random forest, SHAP, risk prediction, mortality, hip fracture, fragility fracture, and postoperative complications. K3 corresponded to imaging and radiomics markers of structural fragility, including computed tomography, radiomics, finite element analysis, cortical bone, vertebral fracture, bone strength, and quantitative computed tomography. K4 represented deep-learning applications for prediction-oriented detection and triage, characterized by terms such as artificial intelligence, deep learning, classification, diagnosis, chest X-ray, bone mineral density, and osteoporotic fractures.

These clusters indicate that the thematic structure of this population-specific dataset is organized around four application pathways: screening and risk stratification, machine-learning-based risk modeling, imaging-derived structural fragility assessment, and deep-learning-assisted prediction-oriented detection or triage.

#### 3.2.4 Hotspot evolution and consistency with the knowledge base

Keyword burst analysis identified several rapidly emerging topics between 2022 and 2025 (**Figure 4D**, **Table 4**). The strongest bursts were observed for “screening,” “fracture,” “osteoporosis,” “hip fracture,” and “fracture risk,” indicating sustained attention to disease burden and clinical risk identification. Technology-related terms, including “deep learning,” “machine learning,” “artificial intelligence,” and “prediction model,” showed more recent burst periods, mainly beginning after 2023, reflecting the increasing prominence of algorithm-driven research. Imaging-related terms such as “computed tomography,” “bone mineral density,” and “dual-energy X-ray absorptiometry” also showed evident bursts, indicating the growing integration of imaging data into fracture prediction research.

**Table 4:** Keyword burst analysis.

Rank	Keyword	Freq.	Burst	Start	End	Peak
1	screening	21	9.1345	2022	2025	2024
2	fracture	68	7.9903	2022	2025	2025
3	osteoporosis	206	7.6195	2023	2025	2025
4	hip fracture	98	7.5153	2023	2025	2024
5	fracture risk	60	7.3451	2023	2025	2024
6	prediction	51	6.9937	2023	2025	2024
7	deep learning	34	6.5634	2024	2025	2024
8	machine learning	107	6.3132	2024	2025	2025
9	computed tomography	20	6.3088	2023	2024	2024
10	artificial intelligence	34	6.1466	2024	2025	2025
11	bone mineral density	67	6.1191	2024	2025	2024
12	dual-energy x-ray absorptiometry	29	5.8739	2024	2025	2024
13	elderly	18	5.8298	2024	2025	2024
14	frax	23	5.5211	2024	2025	2024
15	risk prediction	20	5.0696	2024	2025	2025
16	bone	25	5.0295	2024	2025	2024
17	prediction model	42	4.4760	2025	2025	2025
18	aging	9	4.4068	2023	2023	2023
19	risk factor	21	3.0861	2023	2023	2023
20	vertebral fracture	22	2.8678	2024	2024	2024

This table presents the results of burst detection based on the annual distribution of author keywords. Freq., total keyword frequency; Burst, burst strength; Start and End indicate the time span of the burst period; Peak indicates the year with the highest observed frequency. Burst strength reflects the extent to which the frequency of a keyword increased during a specific period relative to earlier levels. This table is used to identify shifts in research hotspots and emerging directions in AI-based fracture risk prediction.

Cross-mapping between keyword clusters and co-citation clusters was used to assess whether current hotspots were supported by identifiable knowledge bases rather than appearing only as keyword-level signals (**Table 5**). K1 aligned closely with C1, indicating that aging-related screening and conventional fracture-risk assessment remained grounded in FRAX-related risk model frameworks and epidemiological evidence. K2 corresponded strongly to C3, linking machine-learning risk stratification with the methodological AI cluster. K3 showed close consistency with C4, suggesting that imaging and radiomics markers of structural fragility were rooted in earlier evidence on BMD, bone strength, and structural fracture assessment. K4 overlapped with C2 and C3, indicating that deep-learning applications for prediction-oriented detection and triage were connected to both clinical management frameworks and AI methods, although this theme had not yet formed a fully independent knowledge module.

**Table 5:** Consistency between hotspot themes and knowledge base.

Keyword theme	Co-cited clus.	Representative references	Core knowledge base	Consistency
K1: AI-integrated conventional osteoporosis and fracture risk assessment	C1	Kanis JA, 2008 (16); Siris ES, 2004 (13); Johnell O, 2006 (18)	FRAX model development, epidemiological foundation, and absolute fracture risk estimation	High
K2: Machine learning for fracture risk modeling	C3	Breiman L, 2001 (17); Smets J, 2021 (15)	Methodological foundation of random forest and machine-learning algorithms for osteoporosis and fracture prediction	High
K3: Radiomics-based structural features and fracture prediction	C4	Marshall D, 1996 (19); Schuit SC, 2004 (20)	Statistical and structural foundation linking BMD, bone strength, and fracture occurrence	High
K4: Deep learning-assisted fracture prediction and diagnosis	C2/C3	Cosman F, 2014 (14); Smets J, 2021 (15)	Integrated application of clinical management frameworks and AI-based predictive models	Partial

This table presents a cross-analysis of keyword co-occurrence clusters and reference co-citation clusters. Clus., cluster. By matching hotspot themes with corresponding co-cited clusters and representative highly co-cited references, the table evaluates whether major research themes are supported by a stable knowledge base. “High” indicates close correspondence between the hotspot theme and the co-citation structure with clear theoretical or methodological support, whereas “Partial” indicates the presence of relevant support but the absence of a fully independent knowledge block.

Taken together, the integrated evidence from co-citation structure, keyword clustering, burst detection, and hotspot–knowledge-base cross-mapping suggests a layered knowledge system. Established fracture-risk and osteoporosis-screening frameworks provide the clinical foundation, imaging and radiomics expand structural fragility assessment, machine-learning models support risk stratification, and deep-learning applications are moving toward prediction-oriented detection and triage.

### 3.3 Interdisciplinary diffusion pathways and journal-level aggregation

#### 3.3.1 Temporal variation in citing disciplines and thematic diffusion paths

**Figure 5:**
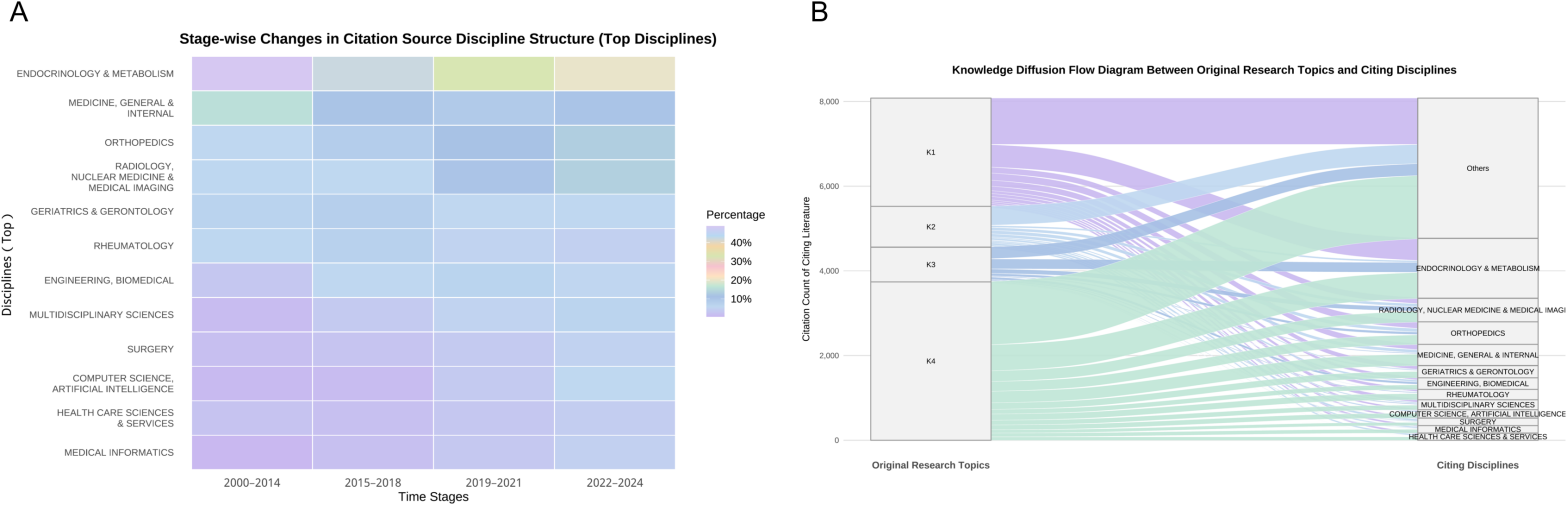
Thematic diffusion patterns across citing disciplines. **(A)** Temporal distribution of citing disciplines, showing changes in disciplinary composition over time. **(B)** Flow structure between major research themes and citing disciplines, illustrating differentiated diffusion paths of thematic knowledge.

To further characterize cross-disciplinary knowledge diffusion, we examined citing disciplines and their flow relationships. The disciplinary composition of citing literature changed over time (**Figure 5A**). In the earlier stage, citations were concentrated mainly in Clinical Medicine and bone-related clinical fields. From approximately 2015 onward, Radiology & Imaging and Engineering became increasingly visible, and after 2019, Computer & AI showed a marked increase. In the most recent period, the proportions of citations from technical disciplines further increased, indicating a gradual expansion of knowledge diffusion from a predominantly clinical base toward broader multidisciplinary participation.

The flow structure between major research themes and citing disciplines showed differentiated diffusion paths (**Figure 5B**). Traditional osteoporosis and fracture risk assessment themes were mainly linked to Clinical Medicine and related clinical fields. Machine learning and predictive modeling showed a dual diffusion pattern, extending into both clinical and technical disciplines, especially Computer & AI and Engineering. Radiomics and structural-feature-based prediction primarily diffused into Radiology & Imaging and Clinical Medicine, whereas deep learning-related themes showed stronger diffusion into Radiology & Imaging and Computer & AI, with relatively less flow into Clinical Medicine. These patterns indicate that clinically oriented themes remained concentrated within medical disciplines, whereas algorithm-driven themes showed broader diffusion into technical domains.

#### 3.3.2 Discipline-level knowledge flow and interdisciplinarity

**Figure 6:**
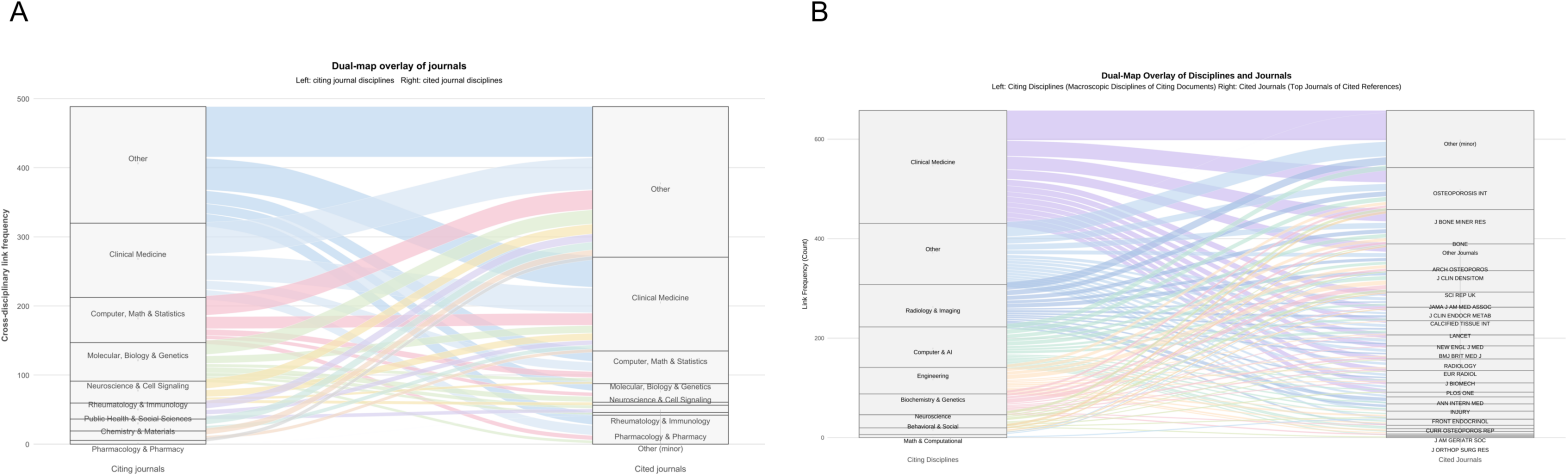
Discipline-level and journal-level knowledge flow. **(A)** Discipline-to-discipline knowledge flow network showing the major cross-disciplinary citation paths. **(B)** Discipline-to-journal flow network showing the major citation destinations of core citing disciplines.

The discipline-to-discipline flow structure showed that Clinical Medicine was the main destination of cited knowledge across the network (**Figure 6A**, **Table 6**). The strongest cross-disciplinary paths were from Radiology & Imaging to Clinical Medicine (516 citations), from Computer & AI to Clinical Medicine (461 citations), and from Engineering to Clinical Medicine (240 citations). These paths accounted for 49.14%, 41.12%, and 42.48% of citations within the corresponding citing disciplines, respectively. Additional flows from Biochemistry & Genetics, Neuroscience, Behavioral & Social, and Math & Computational fields also converged on Clinical Medicine, further indicating its central position in the knowledge structure.

**Table 6:** Core cross-disciplinary knowledge flow matrix.

Rank	Citing field	Cited field	Flow	Global share (%)	Within-field share (%)
1	Radiology & Imaging	Clinical Medicine	516	3.53	49.14
2	Computer & AI	Clinical Medicine	461	3.15	41.12
3	Clinical Medicine	Radiology & Imaging	422	2.88	5.22
4	Engineering	Clinical Medicine	240	1.64	42.48
5	Biochemistry & Genetics	Clinical Medicine	161	1.10	33.68
6	Clinical Medicine	Computer & AI	153	1.05	1.89
7	Neuroscience	Clinical Medicine	153	1.05	46.79
8	Computer & AI	Radiology & Imaging	102	0.70	9.10
9	Behavioral & Social	Clinical Medicine	82	0.56	48.24
10	Math & Computational	Clinical Medicine	45	0.31	48.39

The left column represents the citing disciplines of the source articles, and the upper dimension represents the disciplines of the cited journals in the reference lists. Flow indicates the number of cross-disciplinary citations; Global share indicates the proportion of a given path among all paths; Within-field share indicates its proportion within the corresponding citing field. Only major paths above the preset threshold were retained, whereas low-share merged categories such as “Other (minor)” and “Other (rest)” were excluded. This matrix characterizes the major directions of knowledge input and output across disciplines.

The diversity analysis further supported interdisciplinary integration (**Table 7**). Engineering, Radiology & Imaging, and Computer & AI showed relatively high Shannon diversity values and normalized diversity indices, indicating broader and more balanced cross-disciplinary citation distributions. By contrast, Clinical Medicine showed a comparatively lower normalized diversity index, suggesting a more concentrated citation structure despite its central hub position. Overall, the discipline-level knowledge flow pattern was characterized by a central role of Clinical Medicine, with technical disciplines acting as important bridges in cross-disciplinary knowledge transfer.

**Table 7:** Disciplinary diversity index.

Rank	Citing field	Shannon H	Cited fields (S)	Hmax = ln(S)	Norm. div. (H/Hmax)
1	Pharmacology	0.562	2	0.693	0.811
2	Math & Computational	0.887	3	1.099	0.807
3	Behavioral & Social	0.863	3	1.099	0.786
4	Engineering	1.086	4	1.386	0.783
5	Radiology & Imaging	1.186	5	1.609	0.737
6	Computer & AI	1.183	5	1.609	0.735
7	Neuroscience	1.156	5	1.609	0.718
8	Biochemistry & Genetics	1.091	5	1.609	0.678
9	Clinical Medicine	1.033	5	1.609	0.642
10	Other	1.230	7	1.946	0.632

This table quantifies the diversity of cross-disciplinary knowledge sources for each citing field based on discipline-to-discipline citation relations. Shannon H, Shannon diversity index; S, number of cited fields; Hmax, theoretical maximum diversity under the observed number of cited fields; Norm. div., normalized diversity index. Higher Shannon H values indicate more diverse and dispersed knowledge sources, whereas values of H/Hmax closer to 1 indicate a more even distribution of citations across cited fields and a higher degree of interdisciplinary integration.

#### 3.3.3 Journal-level aggregation of knowledge diffusion

The discipline-to-journal flow structure showed that citations were concentrated in a limited number of core journals (**Figure 6B**, **Table 8**). Clinical Medicine mainly cited Osteoporosis International (947 citations, 32.61% within-field share) and Journal of Bone and Mineral Research (659 citations, 22.69%), followed by Bone and several osteoporosis-related specialty journals. These high-weight paths indicate that journal-level knowledge diffusion was strongly anchored in leading osteoporosis and bone metabolism journals.

**Table 8:** Core discipline-to-journal citation matrix.

Rank	Citing field	Cited journal	Cites	Global share (%)	Within-field share (%)
1	Clinical Medicine	OSTEOPOROSIS INT	947	20.21	32.61
2	Clinical Medicine	J BONE MINER RES	659	14.07	22.69
3	Clinical Medicine	BONE	343	7.32	11.81
4	Other	OSTEOPOROSIS INT	179	3.82	26.28
5	Clinical Medicine	ARCH OSTEOPOROS	146	3.12	5.03
6	Clinical Medicine	J CLIN DENSITOM	137	2.92	4.72
7	Clinical Medicine	JAMA J AM MED ASSOC	126	2.69	4.34
8	Other	J BONE MINER RES	123	2.63	18.06
9	Clinical Medicine	CALCIFIED TISSUE INT	106	2.26	3.65
10	Radiology & Imaging	OSTEOPOROSIS INT	97	2.07	26.22

This table reports the top 10 weighted paths in the discipline-to-journal citation matrix. Cites = total citation counts from a given discipline to a given journal; Global share = proportion of the path among all paths; Within-field share = relative weight of the journal within the corresponding citing field. The table provides quantitative support for the dual-map flow structure shown in **Figure 6**.

Other disciplines also showed aggregation toward the same core journals. For example, citations from Radiology & Imaging to Osteoporosis International accounted for 26.22% of citations within that discipline, and citations from other fields were likewise concentrated in Osteoporosis International and Journal of Bone and Mineral Research. Although general medical journals such as JAMA appeared among the major cited journals, their relative path weights were much lower than those of specialty journals. This pattern indicates a two-level diffusion structure in which knowledge inputs became increasingly multidisciplinary, whereas publication-level consolidation remained anchored in specialized osteoporosis and bone metabolism journals.

## 4 Discussion

### 4.1 Overall research development trends and structural characteristics

Annual, cumulative, and stage-based publication patterns show that AI-related fracture risk assessment, screening, and prediction among middle-aged and older adults have entered a phase of rapid expansion. After 2019, publications increased sharply, with a further acceleration in 2022, and the output of the past three years significantly increased, accounting for a larger proportion of the total sample. Simultaneously, citations have continued to rise, with no sign of plateauing (8, 21). This suggests ongoing diffusion in the field, which is likely related to the rising burden of osteoporotic fractures in aging population (1). The growing demand for early screening and risk stratification has increased interest in AI-supported clinical decision-making (22). Additionally, advancements in machine learning and deep learning for multivariate modeling and imaging feature extraction have promoted the integration of AI into existing risk assessment systems and expanded prediction-oriented application scenarios (23).

The collaboration network exhibits a core-periphery structure, with China and the US as key contributors, while countries like the UK, Canada, and Germany form a secondary tier. This reflects the dependence of fracture risk research on sample size and long-term data, with core countries having advantages in cohort resources and model development (24, 25). Research activity is distributed across several directions, including algorithm development, radiomics-based modeling, and deep learning-based prediction (26, 27). While this fosters rapid technological progress, it also highlights the need for further standardization in cross-center validation and population adaptation.

Overall, the rapid increase in publications and citations suggests growing research activity in AI-based fracture risk assessment, screening, and prediction for middle-aged and older adults. The main contribution of this population-specific bibliometric map is to connect the field’s knowledge base, emerging hotspots, and diffusion pathways within a single structural framework. These patterns should be interpreted as evidence mapping rather than evidence evaluation. Future work should place greater emphasis on aging-specific validation, multimodal data integration, model robustness, and real-world applicability to better support long-term bone health management in middle-aged and older adults.

### 4.2 Knowledge base structure, hotspot evolution, and cross-mapping

The reference co-citation network provides a knowledge-base perspective distinct from that offered by keyword co-occurrence analysis. Rather than only showing current research topics, it identifies the intellectual foundations on which AI-related fracture risk assessment, screening, and prediction in middle-aged and older adults have been built. The highly connected co-citation structure indicates that this population-specific research area is not developing as an isolated AI application field, but remains anchored in accumulated evidence from epidemiology, fracture-risk models, clinical guidelines, BMD-related studies, and emerging machine-learning methods. Four co-citation clusters form a hierarchical gradient in terms of time distribution, size, and total link strength, reflecting the consistent internal logic of knowledge evolution in fracture risk prediction. The earliest average publication year is in C4, which focuses on risk assessment frameworks and epidemiological evidence. This cluster has maintained stable citation levels, highlighting the foundational role of early risk stratification systems in current research. Since fracture risk is a long-term cumulative outcome, it relies on cohort data and probabilistic models. Foundational work such as the development of the QFractureScores algorithm using a cohort of more than 2 million people continues to support AI-based fracture risk research (28, 29). C1, centered on risk prediction models, has a broad scope and long time span, indicating that traditional models like FRAX remain firmly embedded in clinical decision-making (26, 30). AI’s development is enhancing variable integration and complex relationship modeling within existing frameworks (31). C2 revolves around clinical guidelines and standardized management, reflecting the integration of risk assessment research. Guidelines, such as osteoporosis fracture management, play a critical role in connecting evidence with practice and have significant implications for fracture prediction (22, 32, 33). The relationship between C2 and C4 suggests that guideline-based management is grounded in earlier epidemiological and risk-model evidence, providing a standardized framework for model application (34). C3 has the highest link strength and forms dense intersections with the risk model cluster. This structure aligns with the technical demands in fracture risk prediction, as factors like metabolic status, bone structure, and imaging parameters require high-dimensional data and nonlinear modeling. Machine learning methods, like those integrated into models for osteoporotic fracture prediction, have been increasingly explored as approaches for handling these complexities (35, 36). Overall, knowledge evolution follows a hierarchical structure: starting with risk frameworks, supported by evidence, moving through guideline integration, and driven by enhanced methodologies. AI-related research in fracture risk prediction builds on existing clinical evaluation systems, with increasing attention to model development and risk stratification. As image data and multi-source integration increase, knowledge connections may further strengthen around the intersection of methods and models, raising the bar for external validation and cross-population stability.

The author keyword clusters further show how this knowledge base is being translated into application-oriented research themes. Compared with a general hotspot map, the present analysis emphasizes how current keywords are organized around aging-related screening, machine-learning risk stratification, imaging-derived structural fragility assessment, and deep-learning-assisted prediction-oriented detection or triage. This thematic differentiation closely aligns with the multidimensional nature of fracture risk prediction. Fracture risk in the elderly involves both long-term population management and individual structural phenotype and imaging feature assessments, naturally leading to a division of research paths between traditional risk tools, statistical modeling, imaging analysis, and automated recognition. K1 focuses on osteoporosis, screening strategies, and the integration of clinical variables, reflecting that risk prediction research still starts with existing assessment frameworks (37). For the elderly, risk stratification directly impacts screening frequency and intervention decisions, making model operability and interpretability key constraints (38). K2 combines machine learning algorithms with specific outcome indicators and interpretability methods, shifting the research focus from performance comparison to model transparency and clinical applicability, such as developing machine learning-based osteoporosis risk models for clinical decision support (39). K3 integrates imaging features with structural parameters to create a multidimensional risk phenotype, providing new variables for fracture-risk modeling, especially considering how bone quality and structural strength affect fracture risk (40). K4 focuses on deep learning in imaging classification and automated prediction, reflecting the trend toward image-driven screening, such as using X-rays for automated screening of vertebral fractures to improve osteoporosis detection and management (41).

Keyword burst analysis further supports these temporal patterns. Disease and risk-related terms remain active toward the end of the sample, indicating that fracture risk assessment continues to be a central focus (42). Keywords related to algorithms and model construction show bursts in recent years, highlighting methodological upgrades as key drivers of recent growth (43). The cross-mapping between keyword themes and co-citation clusters is a key analytical layer of the present study. It shows that several active research hotspots are not isolated keyword-level phenomena, but are supported by identifiable knowledge bases. For example, aging-related screening and conventional risk assessment themes align with FRAX-related and epidemiological evidence, machine-learning risk stratification aligns with methodological AI foundations, and imaging or radiomics themes are connected with BMD, bone strength, and structural assessment literature. By contrast, deep-learning-assisted prediction-oriented detection and triage shows only partial correspondence with existing co-citation clusters, suggesting that this area remains more dispersed and has not yet consolidated into an independent knowledge base.

Overall, the combined evidence from co-citation structure, keyword clustering, and hotspot–knowledge-base cross-mapping suggests that AI-related research in this population is organized around a layered knowledge system. Established fracture-risk and osteoporosis-screening frameworks provide the clinical foundation, imaging and radiomics expand structural fragility assessment, machine-learning models support risk stratification, and deep-learning applications are moving toward prediction-oriented detection and triage. This layered structure helps clarify how AI research in aging-related bone health deepens and reorganizes existing fracture-risk knowledge rather than simply replacing traditional assessment frameworks.

### 4.3 Knowledge diffusion, interdisciplinary integration, and publication-level aggregation

Compared with simple subject category counts, the flow-based analysis shows not only which disciplines are involved but also how knowledge moves among technical fields, clinical medicine, and specialty journals. The temporal distribution of citing disciplines indicates a phased diffusion path of AI in fracture risk prediction for older adults. Early citation sources were concentrated in clinical disciplines, followed by a rise in Radiology & Imaging and Engineering. Since 2019, Computer & AI has become increasingly prominent, and its share has continued to grow in recent years. This shift appears to be closely related to the data types used in fracture risk prediction research. Early studies mainly relied on population-based cohort data and clinical variables (44, 45). As imaging data became more widely available and high-dimensional feature extraction improved, technical disciplines gradually became major sources of knowledge input, such as studies integrating HR-pQCT-derived structural information into fracture assessment tools and combining electronic health records with radiological imaging to build multimodal deep learning models (46, 47). The maturation of AI methods has therefore strengthened the role of computing-related disciplines in the recent diffusion structure.

The correspondence between themes and disciplines further indicates that different application tasks followed different diffusion paths. Screening and conventional risk assessment themes mainly remained within clinical systems (48), whereas machine-learning risk stratification showed stronger links with both clinical and technical fields (49). Imaging and radiomics themes were concentrated between Radiology & Imaging and Clinical Medicine (50), while deep-learning-related tasks showed stronger connections with imaging and computing fields (51). These patterns suggest that interdisciplinary diffusion was shaped less by AI as a single technological label and more by the underlying data type and clinical task.

The discipline-to-discipline flow structure and core flow matrix show that Radiology & Imaging and Computer & AI contribute the strongest knowledge input paths to Clinical Medicine, with most major flows converging on Clinical Medicine (52, 53). This pattern is consistent with the application-oriented nature of the field, in which many AI-related studies are organized around clinical risk stratification, screening, and decision-support scenarios. For example, calibration and optimization of fracture prediction tools have been examined as approaches to refining risk estimation and supporting patient management (54). Clinical Medicine therefore appears as the major integration hub in the citation network. Although some reverse flows from clinical to technical disciplines are also present, the dominant pattern in the current dataset is convergence toward Clinical Medicine. The diversity index further shows that technical disciplines have more balanced knowledge-source distributions, supporting their bridging role, whereas Clinical Medicine shows a more concentrated citation structure, consistent with its position as a knowledge aggregation endpoint (28, 29).

In summary, AI-based fracture risk prediction in older adults shows a diffusion structure based on multi-source technical inputs, with Clinical Medicine as the main convergence point. The diffusion methods of different themes in this structure align with their data dependencies and task attributes, demonstrating functional differentiation and path layering in interdisciplinary integration within this field.

The discipline-to-journal flow structure further showed that interdisciplinary knowledge inputs were ultimately concentrated in a limited number of publication platforms. Clinical Medicine has a primary citation flow towards core journals in osteoporosis and bone metabolism, such as Osteoporosis International and Journal of Bone and Mineral Research (55, 56). The leading paths carried substantial weight both in the global network and within the corresponding discipline, indicating that AI-related research is primarily disseminated and consolidated within specialized journals. Radiology & Imaging and other technical or mixed disciplines also showed visible flows toward these same specialty journals, suggesting that imaging-based and algorithm-related studies are often consolidated within clinically oriented bone health platforms rather than dispersed evenly across technical journals (57). The development of AI in this field revolves around risk assessment and management systems, where model validation is needed within specific populations and clinical decision-making frameworks. For example, Kong et al. validated machine learning models for predicting hip fracture risk in a specific cohort (58). This may explain why knowledge dissemination tends to concentrate in specialized clinical journal. This journal-level concentration suggests that algorithmic and imaging-based studies are still largely evaluated through osteoporosis- and fracture-oriented clinical platforms. For future research, this structure highlights the need to connect technical model development with aging-specific validation, transparent reporting, and real-world screening or referral pathways.

This aggregation effect at the publication platform level aligns with the methodological integration observed in the co-citation structure. AI methods and risk assessment systems form a structural integration, with knowledge organization centered on clinical risk stratification and management needs. The concentration of publication platforms further emphasizes that knowledge diffusion in this field is directed towards clinical applications. For future research, this structure means that external validation, integration of real-world data, and cross-center scalability, and transparent reporting will be important for evaluating the translational value of AI-based fracture risk assessment, screening, and prediction (38, 59). Emerging work on large language models for osteoporosis referral triage also suggests a possible shift from individual risk estimation to workflow-level decision support. Such applications still require prospective validation, safety assessment, calibration, and real-world clinical evaluation before routine use (60). Sustained validation on clinical platforms may help AI models develop more stable translation pathways in fracture risk assessment, screening, and prediction among middle-aged and older adults.

### 4.4 Relationship to recent reviews and clinical translation

The present findings are broadly consistent with recent application-oriented reviews showing that AI applications in osteoporosis are expanding from diagnostic support toward broader clinical workflows involving bone mineral density assessment, imaging-based screening, fracture risk prediction, personalized management, and clinical decision support (10–12). Our bibliometric results provide a structural explanation for this trend. The recent bursts of “deep learning,” “machine learning,” “artificial intelligence,” “bone mineral density,” “computed tomography,” “dual-energy X-ray absorptiometry,” and “prediction model” indicate that current research activity is increasingly concentrated around imaging-based screening, algorithmic risk stratification, and prediction-oriented decision support.

At the same time, this bibliometric evidence map adds a perspective that is not the main focus of narrative or application-oriented reviews. By integrating co-citation networks, keyword clusters, hotspot–knowledge-base consistency, and discipline-to-journal knowledge flows, this study shows that AI-related osteoporosis and fracture-risk research is not developing as a single homogeneous topic. Established fracture-risk assessment and osteoporosis-screening frameworks provide the clinical foundation, imaging and radiomics expand structural fragility assessment, machine-learning models support risk stratification, and deep-learning applications are moving toward prediction-oriented detection and triage. This interpretation is consistent with recent application studies showing the use of automated CT-based deep learning for osteoporosis assessment and the integration of radiomics with clinical data for vertebral fracture risk prediction (40, 53). This structural mapping helps explain how recent AI applications are being incorporated into existing endocrine bone health and fracture-prevention knowledge systems.

From a translational perspective, the expansion of AI-based osteoporosis and fracture-risk research should be interpreted with caution. Before AI tools can be widely incorporated into routine endocrine and bone health practice, several challenges remain to be addressed. Many models still require external validation across age groups, sex, ethnicity, imaging protocols, devices, and healthcare systems. Model calibration, interpretability, fairness, and transparent reporting are also essential for clinical trust and safe decision-making. In addition, prospective multicenter studies and real-world implementation studies are needed to determine whether AI-based screening or prediction tools can improve referral pathways, treatment decisions, fracture prevention, and patient outcomes. This need is illustrated by external validation work on fracture-risk prediction models and emerging studies of AI-assisted osteoporosis referral triage, which both highlight that model performance must be tested in clinically meaningful settings before routine use (38, 60). Future research should therefore move beyond algorithm development and place greater emphasis on clinically validated, interpretable, and workflow-compatible decision-support systems.

## 5 Limitations

This study relied on English-language literature from WoSCC and PubMed. Although WoSCC provides standardized citation metadata suitable for bibliometric network construction and PubMed improves biomedical coverage, the exclusion of Scopus, Embase, non-English literature, and regional databases may have led to incomplete retrieval of relevant publications and may underestimate contributions from some countries, institutions, or specialty communities. Citation-based analyses may be affected by citation bias, time-lag bias, and database indexing bias, especially because recently published studies may not yet have accumulated sufficient citations. Moreover, WoSCC Topic Search and PubMed MeSH/Title/Abstract searches rely on different indexing systems, which may influence retrieval sensitivity and specificity. Although we harmonized the searches at the conceptual level and applied the same eligibility criteria to all retrieved records, some database-specific retrieval differences may remain.

Keyword analysis is influenced by authors’ phrasing habits, synonyms and indexing practices, which could lead to term drift, even after merging terms. To minimize the risk of misinterpreting hotspots based solely on keywords, this study cross-validated results using co-citation structures and the classification of highly co-cited literature. Although the threshold-based sensitivity analyses showed that the major co-citation modules were broadly stable across thresholds of 8, 10, and 12 citations, bibliometric networks and clusters may still be influenced by threshold settings, clustering algorithms, citation accumulation, and time-lag effects. Recently published studies and niche but clinically relevant studies may therefore be underrepresented in the core co-citation network. Future research could extend this map by integrating additional databases, non-English literature, and regional sources. Subsequent evidence syntheses should move beyond bibliometric mapping to evaluate model-level evidence, including external validation, calibration, bias risk, interpretability, fairness, and clinical utility. Prospective multicenter and real-world implementation studies are also needed before AI-based fracture risk assessment, screening, and prediction can be routinely integrated into clinical pathways.

## 6 Conclusions

This study provides a population-specific bibliometric evidence map of AI applications in osteoporosis screening and fracture risk prediction among aging populations. The field has expanded rapidly and is shifting from conventional risk assessment toward machine learning, deep learning, radiomics, imaging-based screening, and more interdisciplinary forms of integration. These findings provide a structured overview of current research activity, but should be interpreted as evidence mapping rather than direct evidence of model performance or clinical effectiveness. Future studies should prioritize multimodal data integration, external validation, calibration, interpretability, fairness, prospective clinical evaluation, and real-world implementation before AI-based tools can be routinely incorporated into endocrine and bone health practice.

## Data Availability

Publicly available datasets were analyzed in this study. This data can be found here: The datasets analyzed in this study were derived from third-party bibliographic databases, including the Web of Science Core Collection and PubMed. The original bibliographic records are searchable through these databases, subject to their respective terms of use and access restrictions. The search strategies, eligibility criteria, and screening process are described in the article. The processed data used for analysis are available from the corresponding author upon reasonable request.
