# Supplementary material for: Artificial intelligence in osteoporosis screening and fracture risk prediction among aging populations: a bibliometric evidence map
Source: Front Endocrinol (Lausanne). 2026 Aug 4;17:1899276. doi: 10.3389/fendo.2026.1899276 (PMC13481210; doi:10.3389/fendo.2026.1899276)
Supplement: Supplementary file 1 [file Table1.docx]

**Supplementary Tables S1. Conceptual harmonization of WoSCC and PubMed search strategies.**

| **Conceptual block** | **WoSCC Topic Search terms** | **PubMed terms** | **Harmonization rationale** |
| --- | --- | --- | --- |
| Bone health and fracture conditions | osteoporosis; bone fracture; fragility fracture; hip fracture | osteoporosis; bone fracture; fragility fracture; hip fracture | Same disease and fracture-related scope |
| Risk assessment and prediction | fracture risk; prediction; prognosis; risk model; risk assessment | fracture risk; prediction; prognosis; risk model; risk assessment | Same prediction-oriented scope |
| AI-related methods | artificial intelligence; machine learning; deep learning; algorithm; neural network; random forest; support vector machine | artificial intelligence; machine learning; deep learning; algorithm; neural network; random forest; support vector machine | Same AI and computational-method scope |
| Aging-related population | aging; elderly; older adult; geriatric; age | aging; aged; elderly; older adult; geriatric | Same population-oriented scope; PubMed used MeSH where appropriate and Title/Abstract terms for non-MeSH expressions |

*Note.* This table summarizes the conceptual harmonization between the WoSCC and PubMed search strategies. The two database searches were organized around the same four conceptual blocks: bone health and fracture conditions, risk assessment and prediction, AI-related methods, and aging-related populations. WoSCC terms were searched using Topic Search, whereas PubMed terms combined MeSH terms where appropriate with Title/Abstract terms for newer, method-specific, or non-indexed expressions. The table is intended to show conceptual consistency between the two searches rather than field-level equivalence between WoSCC and PubMed.

**Supplementary Table S2. Database overlap, deduplication, and eligibility screening by source.**

| **Step** | **Records, n** | **Description** |
| --- | --- | --- |
| Records retrieved from WoSCC | 819 | Records identified through WoSCC Topic Search |
| Records retrieved from PubMed | 63 | Records identified through PubMed search |
| Total records before deduplication | 882 | Combined records from both databases |
| Cross-database duplicates | 56 | PubMed records overlapping with WoSCC records |
| Within-WoSCC duplicate | 1 | Duplicate WoSCC record caused by early access/formal publication record |
| Total duplicates removed | 57 | Removed before eligibility screening |
| Records after deduplication | 825 | Unique records for title, abstract, and bibliographic screening |
| PubMed-only records after deduplication | 7 | Unique PubMed records screened for eligibility |
| PubMed-only records excluded after screening | 7 | Excluded because they did not meet the predefined eligibility criteria |
| Final included publications | 371 | Publications included in bibliometric analysis |

*Note.* This table summarizes database overlap, deduplication, and source-specific screening. The initial search retrieved 819 records from WoSCC and 63 records from PubMed, yielding 882 records before deduplication. Among the PubMed records, 56 overlapped with WoSCC records and 7 were PubMed-only records. One additional duplicate record was identified within WoSCC. After removing 57 duplicate records, 825 unique records remained for eligibility screening. The 7 PubMed-only records were screened using the same predefined eligibility criteria and were excluded because they did not meet the final inclusion criteria. Ultimately, 371 publications were included in the bibliometric analysis.

**Supplementary Table S3. Sensitivity analysis of reference co-citation thresholds.**

| **Co-citation threshold** | **Cited references retained, n** | **Share of all cited references, %** | **Links, n** | **Total link strength** | **Clusters, n** | **Interpretation** |
| --- | --- | --- | --- | --- | --- | --- |
| ≥8 | 110 | 1.01 | 2349 | 3852 | 4 | Broader network; the four major knowledge-base modules were retained |
| ≥10 | 74 | 0.68 | 1386 | 2478 | 4 | Main analysis; balanced network interpretability and core knowledge-base retention |
| ≥12 | 49 | 0.45 | 703 | 1381 | 4 | Stricter core network; the four-module structure remained identifiable |

*Note.* This table reports the sensitivity analysis of the reference co-citation network using alternative citation thresholds for cited references. The main analysis used a threshold of at least 10 citations. Additional analyses were performed using thresholds of at least 8 and at least 12 citations. For each threshold, the number and proportion of retained cited references, links, total link strength, number of clusters, and overall interpretation were compared. The persistence of four clusters across the three thresholds suggests that the main co-citation structure was broadly stable, although lower-cited recent or niche studies may still be underrepresented in co-citation-based core networks.

**Supplementary Table S4. Author keyword normalization dictionary used for co-occurrence analysis.**

| **Original keyword or variant** | **Normalized keyword** | **Rule** |
| --- | --- | --- |
| bmd | bone mineral density | Abbreviation expansion |
| bone mineral density (bmd) | bone mineral density | Abbreviation harmonization |
| ct | computed tomography | Abbreviation expansion |
| dxa | dual-energy x-ray absorptiometry | Abbreviation expansion |
| qct | quantitative computed tomography | Abbreviation expansion |
| cnn | convolutional neural network | Abbreviation expansion |
| artificial neural networks | artificial neural network | Singular/plural normalization |
| support vector machines | support vector machine | Singular/plural normalization |
| machine-learning | machine learning | Hyphenated/non-hyphenated harmonization |
| deep-learning | deep learning | Hyphenated/non-hyphenated harmonization |
| support-vector machine | support vector machine | Hyphenated/non-hyphenated harmonization |
| neural-network | neural network | Hyphenated/non-hyphenated harmonization |
| fractures | fracture | Singular/plural normalization |
| hip fractures | hip fracture | Singular/plural normalization |
| vertebral fractures | vertebral fracture | Singular/plural normalization |
| ovcf | osteoporotic vertebral compression fracture | Abbreviation expansion |
| type 2 diabetes | type 2 diabetes mellitus | Terminology harmonization |
| older adults | elderly | Population-term harmonization |
| older people | elderly | Population-term harmonization |
| aged | elderly | Population-term harmonization |
| prediction models | prediction model | Singular/plural normalization |
| predictive model | prediction model | Terminology harmonization |
| predictive models | prediction model | Terminology harmonization |
| clinical prediction model | prediction model | Terminology harmonization |
| predictive modeling | prediction model | Terminology harmonization |
| risk factors | risk factor | Singular/plural normalization |
| bones | bone | Singular/plural normalization |
| bone density | bone mineral density | Terminology harmonization |

*Note.* This table lists the main author keyword variants and normalization rules applied before keyword co-occurrence analysis. Normalization included abbreviation expansion, abbreviation harmonization, singular/plural normalization, terminology harmonization, and population-term harmonization. Terms were merged only when they referred to the same concept or to a clearly equivalent variant of the same concept. Related but conceptually distinct terms were retained separately to reduce the risk of over-merging. Non-thematic terms introduced during export or parsing were removed before analysis.
